# Personal

**Published:** 1854-06

**Authors:** N. S. Davis


### EDITORIAL.

*Personal.*

I the May Number of the Journal Prof Herrick announced that he had transferred his interest, as one of its Editors and Proprietors, to me. In formally assuming the duties and responsibilities of editorial life, I assume no new or untried relation Neither am I so much a stranger to the patrons of the *North Western Medical and Surgical Journal*, that any explanations or promises are required in relation to the future The *Journal* from its commencement has been under the charge of able and distinguished men. Any promises on my part to do better than they have done, would be presumptious and unnecessary. I shall certainly do my best, in connection with my colleague Dr Johnson not only to maintain the past reputation of the *Journal*, but also to enlarge and improve it, with the constantly advancing interests and wants of the profession in the North-West One thing, however, I will unhesitatingly promise; from various causes, the last few numbers of the *Journal* have been issued considerably behind the date of publication, and we fear some of the subscribers did not get the May number until the last week in June. Commencing thus behind time it will take perhaps two numbers to catch up; but hereafter, the *Journal* will be issued more promptly during the first week in each month, or my Editorial connection with it will be very brief. It is our intention to make the *Journal* a valuable, if not indispensible aid to the practitioners of the North-West. In order to do this, we need contributions from practitioners in every section of the country.

Send us then, brethren, the results of your experience, and observations in reference to any and all the diseases that may come under your notice. Send us also accounts of the doings of your County and local Medical Societies. A good Monthly Medical Journal needs a great variety of matter for its pages. If it has many contributors, the variety is readily kept up; but it is hard to coin an unending supply out of one or two brains, and that too, in the midst of an unceasing round of professional duties.

Hoping for a more intimate acquaintance with the readers of the *Journal*, I remain their humble servant,

N. S. DAVIS.

